# Supplementary material for: Factors influencing survival outcomes in patients with stroke at three tertiary hospitals in Zimbabwe: A 12-month longitudinal study
Source: PLoS One. 2024 Dec 30;19(12):e0302244. doi: 10.1371/journal.pone.0302244 (PMC11684730; doi:10.1371/journal.pone.0302244)
Supplement: Attachment — Submitted filename: Responses to Reviewers.pdf [file pone.0302244.s003.pdf]

Rebuttal Letter

Dear Editors

Please find the responses to the comments raised by academic editor and reviewers

REVIEWER COMMENTS	RESPONSES TO QUESTIONS
**Journal requirements:**	
1. Please ensure that your manuscript meets PLOS ONE's style requirements, including those for file naming. The PLOS ONE style templates can be found at; https://journals.plos.org/plosone/s/file?id=wjVg/PLOSOne_formatting_sample_main_body.pdf and https://journals.plos.org/plosone/s/file?id=ba62/PLOSOne_formatting_sample_title_authors_affiliations.pdf	Done
2. Please include a complete copy of PLOS' questionnaire on inclusivity in global research in your revised manuscript. Please find more information on the policy and a link to download a blank copy of the questionnaire here: https://journals.plos.org/plosone/s/best-practices-in-research-reporting. Please upload a completed version of your questionnaire as Supporting Information when you resubmit your manuscript.	Included it
3. We note that you have provided funding information that is currently declared in your Funding Statement. However, funding information should not appear in the Acknowledgments section or other areas of your manuscript. We will only publish funding information present in the Funding Statement section of the online submission form. Please remove any funding-related text from the manuscript and let us know how you would like to update your Funding Statement.<br><br>Currently, your Funding Statement reads as follows:	Deleted the funding information from the Acknowledgements section and included it in the cover letter

"Farayi Kaseke was supported by the Training for Research Excellence and Mentorship in Tuberculosis (TRENT) Program, Grant Number D43TW009539. from the National Institute of Health (NIH) as a PhD scholar. This grant was awarded in collaboration with the University of Zimbabwe Faculty of Medicine. The funding was used for data collection purposes. The funding grant ended on 31st December 2023."<br><br>Please include your amended statements within your cover letter; we will change the online submission form on your behalf.	
4. In the online submission form, you indicated that [Data is available upon request.].<br>All PLOS journals now require all data underlying the findings described in their manuscript to be freely available to other researchers, either 1. In a public repository, 2. Within the manuscript itself, or 3. Uploaded as supplementary information.<br>This policy applies to all data except where public deposition would breach compliance with the protocol approved by your research ethics board. If your data cannot be made publicly available for ethical or legal reasons (e.g., public availability would compromise patient privacy), please explain your reasons on resubmission and your exemption request will be escalated for approval.	Data was uploaded as a supplementary file (Excel file)
5. Please include a separate caption for each figure in your manuscript.	Done
**Reviewer #1: Overall**	
Introduction<br>The background provides a clear rationale for the study. It might benefit from expanding on why these particular determinants were added (Line 72-73).	This was added: (Line 82-87). Hospital bill payment modalities can reveal economic barriers and financial stress that affect recovery trajectories post-treatment(1,2). Educational attainment may influence health literacy, access to healthcare resources, and ultimately, health outcomes(3,4). Housing status is linked to social support levels and environmental stability, both of which are pivotal for long-term recovery and well-being(5). Total functional outcomes provide a measure of recovery, encompassing physical, cognitive, and social dimensions(6). This study sought …
Methods<br>However, it would be helpful to include more information on the validation of the data collection tools and potential sources of bias.	This was added: (Line 158-159). The data collection tool was validated for reliability before deployment. The Functional Independence Measure (FIM™) also used in this study, was validated and found to be a reliable tool for stroke patients(7–9).

Results and Discussion	No issues raised
Specific<br>Heading - The heading could benefit from being more specific. Consider including that this study was conducted in three tertiary hospitals to provide clearer context.	Topic rephrased to: **Factors influencing survival outcomes in patients with stroke at three tertiary hospitals in Zimbabwe: A 12-month longitudinal study**
Abstract - The first hazard ratio (HR) mentioned in the abstract may need to be written out in full, and then subsequent hazard ratios can be abbreviated to maintain clarity.	This was corrected: (Line 46). (Hazard Ratio; HR: 0.4, 95% CI: 0.20, 0.80).
Sample Size - There was a 27% loss in the sample size. It would be helpful to know if this was accounted for in the initial sample size calculation and its potential impact on the study's statistical power.	This was added: Line 102-105). The initial sample size calculation accounted for anticipated attrition rates, to maintain the study's statistical power despite the loss. This consideration was to ensure that the conclusions drawn were based on a more homogeneous and complete dataset. However, the attrition rate ended up being higher that had been anticipated and this was a limitation of the study.
Exclusion Criteria (Line 97-96) - Providing a rationale for excluding Transient Ischaemic Attacks (TIA) would strengthen the clarity and justification of the exclusion criteria.	Rephrased: Line 119-121). The study excluded admitted patients who died before medical stabilization or refused to participate, as well as those who had a Transient Ischaemic Attack (TIA). The rationale for excluding TIA cases was to ensure the study population consisted of patients with more definitive and severe cerebrovascular events, thereby reducing variability and improving the precision of the study's outcomes.
Operational Definitions - Including a definition of stroke would be beneficial. Was the study looking at all types of Stroke or just Ischaemic Strokes?	This was added under operational definitions (Line 139-151). **Stroke:** Stroke was defined as a focal neurological deficit caused by a disturbance of the circulation to the brain following an acute or sudden disturbance of brain function vascular in origin causing disability lasting more than, or death within 24 hours. The study included both ischaemic and hemorrhagic strokes.
Data Collection Process - Was the principal investigator present to conduct data quality checks upon collection by the research assistants? Clarifying this would ensure confidence in data integrity.	This was added: (Line 156-157). The principal investigator was present to conduct data quality checks upon collection by the research assistants.
Variables<br>- Under hospital bill modalities, how does 'Free Service' differ from 'Social Welfare'?<br>- How was the total function of the patients measured? Provide more detail on this measurement to enhance understanding.	"Social welfare" was combined with "free service" since both categories represent services provided at no direct cost to individuals. This will reflect 16.06% of the population benefiting from the support mechanisms. Appropriate changes were made in the manuscript.

	This was further added to the FIM definition under data collection procedure: Total score for FIM was measured as the sum of the motor and cognition subscale scores.
Results Table - The results table could benefit from including specific p-values for the important variables to give readers a clearer sense of statistical significance.	P-values were added on Table 1 and 3.
Discussion (Line 60-62) - The Thailand study mentions contrasting results, what are the Author's thoughts on this. Could these differences be due to variations in study design? Expanding on this shows critical evaluation of the paper.	This was added in discussion section: The differences could potentially stem from variations in study design, including differences in patient demographics, healthcare systems, treatment protocols, and follow-up durations.
Discussion (Line 67-68) - There are minor spelling errors ("worsen" and "further") that need correction.	Original: Thus once they suffered a stroke, their conditions got worsen being furthers compounded by inability to travel or fund medications<br>Revised: Thus once they suffered a stroke, their conditions worsened, further compounded by the inability to travel or fund medications : 94-95
Discussion (Line 77) - The statement that higher educational attainment "correlated" with a reduced risk of death needs to be substantiated. The findings indicate that secondary education had a higher survival rate, but the results for primary and tertiary education were not significant in the Gompertz AFT model. Survival status in Table 1 also shows a higher survival status in those with secondary compared to tertiary education. Thus, perhaps reconsideration of the word 'correlated' is needed.	Revised: Educational attainment emerged as a significant predictor of mortality post stroke, unveiling a trend where a reduced risk of death was observed among those with higher education. Particularly noteworthy was the significantly lower risk observed in individuals with secondary school education compared to those with primary education….
Discussion (Line 92-96) - The impact of housing status could be confounded by the presence of a support system. The conclusion stated is about support system, however the data collected is on housing status alone. Clarify whether the study is assessing the effect of having one's own house or the presence of relatives in the house.	Revised: Housing status emerged as another determinant, revealing a protective effect for those living with parents/relatives. This suggests that the presence of relatives in the house may offer a supportive environment that mitigates mortality risk. This implies that the observed protective effect could be influenced by the support system inherent in living with family, rather than housing status alone.
Rehabilitation Attendance - The study could benefit from assessing trends in rehabilitation attendance post-discharge. If data on this is available, reporting it would add depth to the findings.	All the patients were presumed to have been receiving the usual rehabilitation care but unfortunately, data on rate of rehabilitation attendance post-discharge were not available for analysis in this study.
Hospital Payment Modalities - What were the specific p-values for health insurance and social welfare? If these values are between 0.05 and 0.1, it could strengthen your argument that these variables warrant further exploration.	The p-values were incorporated into the analysis as recommended. "Social Welfare" was combined with "Free Service" as both categories represent services provided at no direct cost to individuals, collectively reflecting 16.06% of the population benefiting from these support mechanisms. This

	consolidation was to simplify analysis and enhance the clarity of the impact of cost-free services.
Strengths and Limitations - The statement that recruitment from central hospitals improves generalisability may need reconsideration. Patients at central hospitals are often referred for advanced care, which may not represent the broader population.	This statement was removed.
**Reviewer #2**	
Editorial/English language revision required; typos, sentencing and the like needs to be thoroughly revised; please may respect be payed to grammar and spelling. A manuscript of this prospected calibre should be devoid of major errors in language (page 3, line 63/64: "necessitate the need" - this is atrocious English...).	Revised: Understanding factors influencing survival outcomes, will provide a foundation for targeted interventions customized to the region's unique socio-economic and healthcare landscape.<br>The English language was revised
The study design:<br>It appears that this paper is a sub-analysis of a "larger" (page 4, 86) study which I don't see referred to in reference or text; was the data collected prospectively? it appears so, but is not explicitly stated. Who identified the patients? How were they recruited? This is crucial to the understanding of the message.<br>Do the respective hospitals have stroke units? The three hospitals were manned by neurologists? (page 4,89). I am not aware that this is the case, never mind weekly outpatient stroke clinics for "all" stroke patients discharged...	Revised: This paper is a sub-analysis of a larger study carried out to develop, implement and preliminarily evaluate outcomes of stroke survivors and their caregivers in Harare (13). The data were collected prospectively. The first author and research assistants identified the patients based on the inclusion criteria and consecutive sampling was done with patients recruited as they were admitted from each of the three hospitals.<br>At the time of data collection, only Parirenyatwa Group of Hospitals had a stroke unit. Parirenyatwa and Harare Central Hospitals were manned by neurologists while Chitungwiza hospital was manned by general practitioners and physicians who were not specifically neurologists. Although weekly outpatient stroke clinics for "all" stroke patients discharged are expected at these hospitals, it was no longer possible due to reduced numbers of physiotherapists and occupational therapists manning the out-patient clinics hence patients were seen once or twice a month.
Page 6 and page 8, the Ethics statement is duplicated.	Ethics under data collection procedures was removed, and ethics details were retained under the section for 'Ethics approval and participant consent'.
Results:<br>Page 8:<br>The gross discrepancy in mortality between the three hospitals needs to be explored and further explained: is it different populations with different pathologies, different referral pathways or else? Are these all tertiary referral centres, if so why these stark contrasts?	This likely arises from differences in referral pathways and hospital capabilities. Detailed analysis of referral patterns and the specialized services offered by these hospitals will need to be conducted to understand the variations. This will help to explain the reasons behind the mortality contrasts.

Page 9:<br>86: regarding level of education, only three (3) patients were uneducated (no formal education), all of whom died within the 12 months follow up, obviously making it 100%; this is too small a number to even mention!!!	Well noted, we corrected ‘Education by excluding this category’, which led to slight changes in the Hazard ratios.
Page 10:<br>Comorbidities: saying that 21 patients had no comorbidities; this is from a clinician's point of view very unlikely and hard to believe.<br>Was HIV status known in the cohort or undetermined or ignored? This would have been very valuable info.	Comorbidity was not significant: Variable for ‘comorbidities’ was available, and 96% (131/137) of the participants had other underlying conditions. However, this variable was not eligible for further analysis since its p value was >0.25 at bivariate analysis.<br>HIV data: HIV data were excluded from the manuscript due to a high proportion of missing information (71 cases – 52%). Among these cases, 69.01% were deceased, indicating potential bias in the analysis if imputed or incomplete data were used.
The outcomes/survival times according to payer categories don't make sense as the non-paying ones are better off (but these are older pensioners etc.). The privately paying patients had poorer survival, but this may be related to the fact that these were sicker patients with no choice but biting the "private" bill? People in this category will not go to hospital for minor strokes. Patients staying with parents/relatives have better survival because they are probably younger...	The inclusion of hospital bill payment modalities is crucial as it sheds light on economic barriers and financial stressors that influence recovery, as discussed in the study introduction & discussion). This is often overlooked, particularly within Zimbabwean contexts, and may similarly pertain to other settings worldwide.
Here is the crux of the paper and my main criticism which can be allayed by proper/different statistics being applied:<br>The authors seem to find associations by the Gompertz AFT modelling statistics because Cox bivariate regression had failed to show significant correlations between the variables and time outcomes. The authors derive correlations based on the AFT stats, but seem to mix up association and causation: the fact that people living with parents/relatives have better outcomes than people living in their own homes may be related to the fact that the former are younger than the latter... These patients have a better outcomes because their strokes might be milder and they can "afford" to seek hospitalisation for minor strokes as opposed to others.	We employed Gompertz parametric regression analysis following unsuccessful diagnostics with the Cox proportional hazards model, evidenced by a significant global test result ($p < 0.05$) and systematic deviations observed in the plot of scaled Schoenfeld residuals against transformed time, indicating violations of proportional hazard assumptions. We then evaluated four parametric models using Akaike’s information criterion and graphical diagnostic assessments, and the Gompertz AFT model provided the most suitable fit for our stroke patient dataset. We used this approach to address methodological challenges and ensure robust statistical inference.
In my view this data set should have been processed by a multivariate regression analysis taking into account the multitude of variables involved in this complex setting of societal and economic factors. The authors make too little and simple reading of what the AFT calculations appear to suggest: there are far too many contradictory outcomes explained too simply as cause and effect.	While acknowledging that multivariate regression analysis could have been an alternative approach, survival analysis using AFT models was deemed appropriate for accurately capturing time-to-event outcomes in our stroke patient dataset.

Reviewer #3: Major comments	
1. Table 1 does not show p-values of the comparisons between those who survived stroke versus those deceased.	We added the p-values to Table 1 and 2
2. There is substantial loss to follow-up. Please kindly provide a Table that compares those who were lost to follow-up versus those who stayed in the study for analysis.	Flow chart diagram was added to the list of figures
3. Discussion: A sample size of 137 is not a 'large sample size' to be reported as a strength of the study. Please remove this statement.	Well noted, the statement was extracted.
4. Discussion: The authors should please discuss the reasons for the disparity in stroke fatality of 25% in their previous study juxtaposed against 58% in the current study.	This was added: This disparity with the current study (58%) could be attributed to differences in study populations, such as variations in demographics, comorbidities and stroke severity. These may have contributed to the higher fatality rate observed in the current study. In addition, the changes in healthcare access and quality of care over time might have impacted patient outcomes.
5. Adoukounou et al has reported a meta-analysis of stroke fatality in Africa of 33.2%. Authors should please discuss their findings in the light of this meta-analytic data.	This was done. The pooled CFR for SSA was lower than the 33,2% in the meta-analysis indicating possible poor management of stroke in the three hospitals
6. The use of reference [1] is not correct as this is a Review article. The incidence, prevalence, mortality rates of stroke in Africa have specific studies which should be cited, not the Review article which summarizes these findings.	The authors note this comment, Cited specific studies.

References

1. Beckman AL, Bucholz EM, Zhang W, Xu X, Dreyer RP, Strait KM, et al. Sex differences in financial barriers and the relationship to recovery after acute myocardial infarction. J Am Heart Assoc. 2016;5(10).
2. Soares CLA, Magalhães JP, Faria-Fortini I, Batista LR, Andrea Oliveira Lima L, Faria CDCM. Barriers and facilitators to access post-stroke rehabilitation services in the first six months of recovery in Brazil. Disabil Rehabil. 2024;
3. Jansen T, Rademakers J, Waverijn G, Verheij R, Osborne R, Heijmans M. The role of health literacy in explaining the association between educational attainment and the use of out-of-hours primary care services in chronically ill people: A survey study. Vol. 18, BMC Health Services Research. 2018.
4. Chen P, Callisaya M, Wills K, Greenaway T, Winzenberg T. Cognition, educational attainment and diabetes distress predict poor health literacy in diabetes: A cross-sectional analysis of the SHELLED study. PLoS One. 2022;17(4 April).
5. Eklund M, Argentzell E, Bejerholm U, Tjörnstrand C, Brunt D. Wellbeing, activity and housing satisfaction - comparing residents with psychiatric disabilities in supported housing and ordinary housing with support. BMC Psychiatry. 2017;17(1).

6. Gunn S, Burgess GH. Factors predicting rehabilitation outcomes after severe acquired brain injury in trauma, stroke and anoxia populations: A cohort study. Neuropsychol Rehabil. 2022;32(2).
7. Keith RA, Granger C V., Hamilton BB, Sherwin FS. The functional independence measure: a new tool for rehabilitation. Adv Clin Rehabil. 1987;1.
8. Linacre JM, Heinemann AW, Wright BD, Granger C V., Hamilton BB. The structure and stability of the functional independence measure. Arch Phys Med Rehabil. 1994;75(2).
9. Torres A. Capturing functional independence measure (FIM®) ratings. Rehabilitation Nursing. 2018;43(1).
