# Supplementary material for: Factors influencing survival outcomes in patients with stroke at three tertiary hospitals in Zimbabwe: A 12-month longitudinal study
Source: PLoS One. 2024 Dec 30;19(12):e0302244. doi: 10.1371/journal.pone.0302244 (PMC11684730; doi:10.1371/journal.pone.0302244)
Supplement: Attachment — Submitted filename: Revised_Responses to Reviewers.docx [file pone.0302244.s004.docx]

| **REVIEWER COMMENTS** | **RESPONSE** |
| --- | --- |
| **Reviewer #2** |  |
| The authors have addressed a good many of the points raised in the first set of comments. I particularly acknowledge the comment on the exclusion of HIV status data from this paper; please may the authors include this in the text as a point of notice, since HIV status and stroke do have a correlation and Zimbabwe is a high prevalence environment thus HIV is to be considered as a risk factor. | **HIV data:** Analysis by HIV status was not performed due to a significant proportion of participants with missing HIV status data (71 cases - 52%), but remained included in the analysis of other variables. The exclusion of HIV status was necessary to avoid misleading conclusions, as 69.01% of the cases with missing HIV data were deceased, which could have introduced bias if incomplete or imputed data had been used. – **Added under study variables: line 137-141**  The exclusion of analysis by HIV status, due to the large proportion of participants with missing HIV status data, is a limitation of this study. Future studies should focus on collecting more complete HIV data and analyse its potential impact on stroke outcomes, as HIV infection may significantly influence patient prognosis and recovery. – **Added under Strengths and limitations of the study and or findings: line 450-453** |
| My main issue has still not been addressed, yet I do appreciate some of the comments in the authors' response; I copy here directly: | We thank the reviewer for the thoughtful feedback and for highlighting critical aspects of the study that require further clarification and discussion. We have carefully considered the comments, and we would like to provide a detailed response to address the points raised below. |
| The outcomes/survival times according to payer categories don't make sense as the non-paying ones are better off (but these are older pensioners etc.). The privately paying patients had poorer survival, but this may be related to the fact that these were sicker patients with no choice but biting the "private" bill? People in this category will not go to hospital for minor strokes. | **Survival Times according to payer categories:** We acknowledge the reviewer’s observation that the survival times for non-paying patients appeared to be better, while privately paying patients exhibited poorer survival outcomes. This apparent discrepancy may indeed reflect differences in the severity of illness rather than solely the payment modality. Privately paying patients might be more likely to seek care only when their condition is severe, leading to worse outcomes. On the other hand, non-paying patients, including older pensioners, may have better access to public healthcare services and seek medical attention for milder conditions, which could result in better survival outcomes.  **Discussion section revised to add the following, line 414-430:** The observed differences in survival times across Hospital bill payment modalities may reflect not only economic disparities but also differences in health-seeking behaviors and illness severity at the time of hospital admission. Non-paying patients, which include social welfare, pensioners, retirees, and those older than 65, likely have earlier access to public healthcare services, potentially seeking care for less severe conditions, which could explain their better survival outcomes. Conversely, cash paying patients may delay seeking care due to financial barriers, only presenting at hospitals when their condition becomes critical. This difference in access to timely care likely contributes to the poorer outcomes seen in the privately paying group. This demonstrates the impact of economic barriers on health outcomes, particularly in contexts like Zimbabwe, where financial constraints may limit access to healthcare. The relationship between healthcare payment modalities and survival outcomes warrants further investigation, as it reflects broader socioeconomic inequalities that can significantly influence recovery and long-term survival. |
| The authors derive correlations based on the AFT stats, but seem to mix up association and causation: the fact that people living with parents/relatives have better outcomes than people living in their own homes may be related to the fact that the former are younger than the latter... These patients have a better outcomes because their strokes might be milder and they can "afford" to seek hospitalization for minor strokes as opposed to others. | **Living with parents/relatives and better outcomes:** We agree with the reviewer that patients living with parents or relatives tend to have better survival outcomes, potentially due to their younger age and less severe strokes. Younger patients may be more resilient and have better recovery prospects, and they may also be more likely to seek care for milder strokes. While our model identified this association, we acknowledge that the relationship between living arrangements and stroke outcomes may be influenced by factors such as age and stroke severity, which warrant careful consideration in the interpretation of our findings.  **Discussion section revised to add the following, line 385-397:** The improved outcomes associated with living with family may also be influenced by factors such as younger age and fewer comorbidities among these patients, making them more likely to recover from stroke. In addition, the support provided by family members likely facilitates quicker access to care, better adherence to treatment, and more effective management of overall health. In contrast, patients living independently may delay seeking treatment, contributing to poorer outcomes. While our analysis identified this association, it is important to acknowledge the role that age and stroke severity play in shaping these results. Future research should explore on the impact of family support on stroke recovery, especially in resource-limited settings where informal caregiving is a critical component of patient care. |
| Secondary education is better than primary education, but also better than tertiary education for stroke outcomes? Please may you read through the above comments again and reply accordingly in the manuscript with the arguments raised. | **Education Levels and stroke outcomes:** Regarding the relationship between education levels and stroke outcomes, we recognize the unexpected result where secondary education appeared to be associated with better outcomes than both primary and tertiary education. We propose that this could be explained by several factors, including socioeconomic status, health-seeking behavior, and lifestyle differences between individuals with secondary and tertiary education. Those with secondary education may occupy a socioeconomic position that affords them better access to healthcare and healthier lifestyles, while individuals with tertiary education may face occupational stress and lifestyle factors that negatively affect their health.  **Discussion section revised to add the following, line 365-375:** Interestingly, the better outcomes associated with secondary education, compared to both primary and tertiary education, may be linked to socioeconomic and lifestyle factors. Individuals with secondary education likely occupy a middle socioeconomic tier, which provides better access to healthcare and healthier lifestyles. In contrast, those with tertiary education may face higher levels of occupational stress, contributing to risk factors like hypertension or sedentary behaviour, while individuals with primary education may lack health literacy or access to adequate healthcare, leading to poorer outcomes. This pattern reflects the complex ways in which education interacts with socioeconomic status and health behaviors to influence health outcomes. Understanding these dynamics is critical, particularly in low-resource settings where disparities in education and healthcare access can significantly affect disease outcomes. |
| While I do accept the employment of the Gompertz AFT modelling statistics for the reasons explained by the authors, may some discussion please be devoted to the above. | **Clarification on the use of the Gompertz AFT model and misinterpretation of causation:** We appreciate the reviewer’s acceptance of the Gompertz AFT model. As mentioned in our previous responses, the selection of the Gompertz AFT model was based on thorough diagnostics, which indicated that it provided the best fit for the data. While significant associations were observed, we agree that it is crucial to distinguish between association and causation. The associations identified by the AFT model should not be interpreted as direct causal relationships but rather as indications of patterns within the data. We have been mindful to frame our findings accordingly and will ensure this is clearly articulated in the manuscript.  **Discussion section revised to add the following, line 437-444:** While the Gompertz AFT model identified significant associations between the observed socioeconomic and demographic factors and stroke outcomes, it is essential to emphasize that these associations do not imply direct causation. For example, the observed better outcomes for patients living with parents or relatives may be linked to their younger age or less severe strokes, rather than the living arrangement itself being a direct cause of improved survival. The model was selected based on its superior fit for the data compared to other models; however, like any statistical model, it identifies relationships that need to be considered in the broader clinical and social context. The intent behind employing the Gompertz AFT model was to provide a more precise analysis of time-to-event data, rather than to establish definitive cause-and-effect links. |
| **Reviewer #3: Major comments** |  |
| 1. Authors have addressed all points raised on my initial review except one key issue.  Authors should please provide a Table that compares participants who were lost to the follow-up versus those who completed the study versus those who died.  For those who were lost, are their demographic, clinical and stroke severity indicators significantly similar or dissimilar to those who completed the study vs those who died? This is an important analysis given the significant attrition reported in your study. Thanks | Table 2 with interpretations added: **line 235-254** |
